# Supplementary material for: Combining Time-Dependent Density Functional Theory and the ΔSCF Approach for Accurate Core-Electron Spectra
Source: J Chem Theory Comput. 2022 Nov 16;18(12):7620–9. doi: 10.1021/acs.jctc.2c00817 (PMC9753589; doi:10.1021/acs.jctc.2c00817)
Supplement: Supplementary file 1 — ct2c00817_si_001.pdf [file ct2c00817_si_001.pdf]

List of geometries in the FHI aims format relaxed using SCAN and default Tight basis sets. Units in Angstroms.

Acetone (CH_3CH_3CO)
atom 4.13121948 0.98028284 -0.00238081 O
atom 3.08002499 0.37506841 0.00018569 C
atom 1.75732561 1.10231248 -0.02242284 C
atom 3.04866898 -1.13581055 0.02293861 C
atom 1.72355666 1.77707757 -0.87710833 H
atom 1.68061004 1.71813159 0.87270548 H
atom 0.90216527 0.43161633 -0.05598333 H
atom 2.04023513 -1.54376036 0.05329664 H
atom 3.54672357 -1.51246139 -0.86938264 H
atom 3.61457026 -1.50245691 0.87815155 H

Acetylene (C_2H_2)
atom 0.00000000 -0.00000000 0.59681178 C
atom 0.00000000 -0.00000000 -0.59681178 C
atom -0.00000000 0.00000000 1.65877136 H
atom -0.00000000 0.00000000 -1.65877136 H

Ethylene (C_2H_4)
atom 0.00000000 0.00000000 0.66053689 C
atom -0.00000000 -0.00000000 -0.66053689 C
atom 0.00000000 0.92137328 1.22667257 H
atom 0.00000000 -0.92137328 1.22667257 H
atom 0.00000000 0.92137328 -1.22667257 H
atom 0.00000000 -0.92137328 -1.22667257 H

Methane (CH_4)
atom 0.00000000 0.00000000 0.00000000 C
atom 0.62760000 0.62760000 0.62760000 H
atom 0.62760000 -0.62760000 -0.62760000 H
atom -0.62760000 0.62760000 -0.62760000 H
atom -0.62760000 0.62760000 0.62760000 H

Carbon Monoxide (CO)
atom 0.00000000 0.00000000 0.00143328 C
atom 0.00000000 0.00000000 1.12676672 O

Ethanol (C_2H_5OH)
atom 1.16099560 -0.37014179 -0.00000000 C
atom -0.03347006 0.54399416 0.00000000 C
atom -1.21189550 -0.25586666 0.00000000 O
atom -1.94334070 0.36811271 -0.00000000 H
atom 2.08999898 0.19940372 -0.00000000 H
atom 1.14576404 -1.00410958 0.88408474 H
atom 1.14576404 -1.00410958 -0.88408474 H
atom 0.00679180 1.20230850 0.87904501 H
atom 0.00679180 1.20230850 -0.87904501 H

Water (H_2O)
atom -0.00000000 -0.00000000 0.09042791 O
atom -0.00000000 0.74982056 -0.51441396 H
atom -0.00000000 -0.74982056 -0.51441396 H

Formaldehyde (HCHO)
atom 0.00000000 -0.00000000 1.20431663 O
atom -0.00000000 0.00000000 0.00465945 C
atom 0.00000000 0.93457758 -0.58958804 H
atom 0.00000000 -0.93457758 -0.58958804 H

Hydrogen Fluoride (HF)
atom 0.00000000 0.00000000 0.00822394 F
atom 0.00000000 0.00000000 0.90857606 H

Ammonia (NH3)
atom -0.00000000 -0.00002470 0.01144920 N
atom 0.00000000 -0.93211217 -0.38540997 H
atom 0.80722010 0.46611844 -0.38541961 H
atom -0.80722010 0.46611844 -0.38541961 H

Carbonyl Sulfide (OCS)
atom 4.10525819 0.71548893 -0.00000000 S
atom 1.74418600 -0.64772048 -0.00000000 O
atom 2.74855581 -0.06776845 0.00000000 C

Pyrazine($C_4H_4N_2$)
atom -0.00000000 -0.00000000 1.42429855 N
atom -0.00000000 0.00000000 -1.42429855 N
atom -0.00000000 1.11949799 0.69773794 C
atom 0.00000000 -1.11949799 0.69773794 C
atom 0.00000000 -1.11949799 -0.69773794 C
atom 0.00000000 1.11949799 -0.69773794 C
atom -0.00000000 2.05613678 1.24401683 H
atom 0.00000000 -2.05613678 1.24401683 H
atom -0.00000000 -2.05613678 -1.24401683 H
atom 0.00000000 2.05613678 -1.24401683 H

Pyridazine ($C_4H_4N_2$)
atom -0.00000000 0.68552134 1.16848867 C
atom 0.00000000 -0.68552134 1.16848867 C
atom 0.00000000 -1.30909248 -0.07879386 C
atom 0.00000000 1.30909248 -0.07879386 C
atom -0.00000000 1.25896168 2.09046166 H
atom -0.00000000 -1.25896168 2.09046166 H
atom 0.00000000 -2.39045835 -0.13145030 H
atom 0.00000000 2.39045835 -0.13145030 H
atom 0.00000000 0.67332310 -1.25712116 N
atom -0.00000000 -0.67332310 -1.25712116 N

Pyridine (C_5H_5N)
atom 0.00000000 0.00000000 1.42915239 N
atom 0.00000000 -0.00000000 -1.38185016 C
atom 0.00000000 1.13455299 0.71985335 C
atom -0.00000000 -1.13455299 0.71985335 C
atom -0.00000000 1.18815506 -0.66969637 C
atom 0.00000000 -1.18815506 -0.66969637 C
atom -0.00000000 0.00000000 -2.46466833 H
atom 0.00000000 2.05132872 1.29569979 H
atom -0.00000000 -2.05132872 1.29569979 H
atom -0.00000000 2.14227385 -1.18007372 H
atom 0.00000000 -2.14227385 -1.18007372 H

Pyrimidine ($C_4H_4N_2$)
atom 0.00000000 0.00000000 1.33794302 C
atom 0.00000000 -0.00000000 -1.30252538 C
atom -0.00000000 1.18130692 0.61391736 C
atom -0.00000000 -1.18130692 0.61391736 C
atom -0.00000000 1.20447284 -0.72127822 N
atom -0.00000000 -1.20447284 -0.72127822 N

atom 0.00000000 -0.00000000 2.42151210 H
atom 0.00000000 0.00000000 -2.38317458 H
atom -0.00000000 2.13997384 1.12252278 H
atom 0.00000000 -2.13997384 1.12252278 H
